# Factors influencing LGBTQ+ disclosure decision-making by Canadian health professionals: A qualitative study

**DOI:** 10.1371/journal.pone.0280558

**Published:** 2023-02-01

**Authors:** Brenda L. Beagan, Kaitlin R. Sibbald, Stephanie R. Bizzeth, Tara M. Pride

**Affiliations:** 1 School of Occupational Therapy, Dalhousie University, Halifax, Nova Scotia, Canada; 2 Dartmouth General Hospital, Dartmouth, Nova Scotia, Canada; Universita degli Studi della Campania Luigi Vanvitelli, ITALY

## Abstract

Disclosure of LGBTQ+ identities at work may reap benefits, but may also exacerbate harms. Faced with ambiguous outcomes, people engage in complex concealment/disclosure decision-making. For health professionals, in contexts of pervasive heteronormativity where disclosure to patients/clients is deemed to violate professional boundaries, stakes are high. This qualitative study with 13 LGBTQ+ health professionals across Canada used semi-structured interviews to explore factors affecting disclosure decision-making, particularly attending to power structures at multiple levels. Most participants engaged in constant risk-benefit assessment, disclosing strategically to colleagues, rarely to clients/patients. At the individual level they were affected by degree of LGBTQ+ visibility. At the institutional level they were affected by the culture of particular professional fields and practice settings, including type of care and type of patients/clients, as well as colleague interactions. Professional power–held by them, and held by others over them–directly affected disclosures. Finally, intersections of queer identities with other privileged or marginalized identities complicated disclosures. Power relations in the health professions shape LGBTQ+ identity disclosures in complex ways, with unpredictable outcomes. Concepts of professionalism are infused with heteronormativity, serving to regulate the gender and sexual identity expression of queer professionals. Disrupting heteronormativity is essential to forge more open professional cultures.

## Introduction

Conservative cultures within the health professions, coupled with intense power hierarchies and pervasive heteronormativity, can make them risky places for learners and professionals to disclose LGBTQ+ (or queer) sexual and gender identities [1, 2]. While disclosure of concealable, stigmatizable identities is often linked with personal and social benefits, it may also lead to increased hostility and persecution [3]. Given the ambiguous outcomes, in work settings queer people may become adept at calculating risk and benefit, employing selective identity disclosure as a skilled coping strategy [4]. Heteronormativity–the dominant ideological stance that heterosexuality is natural and normal, that gender is binary and neatly mapped onto biological sex–thus becomes a force regulating the embodiment and expression of sexual and gender identities, and a particularly harmful normative force for those who identify as LGBTQ+. Given the health disparities and barriers to quality care faced by LGBTQ+ patients [5–7], it is particularly problematic if health professionals who also identify as LGBTQ+ struggle to bring those identities into their work. There is need for nuanced exploration of the complex factors that shape disclosure decision-making [8], particularly taking into account intersections with power structures on multiple levels [9]. In this paper we explore the experiences of 13 LGBTQ+ Canadian health professionals (physicians, nurses and occupational therapists), examining the factors that affect disclosure/concealment decision-making.

### LGBTQ+ workplace concealment/disclosure: Influencing factors

Even in settings with strong human rights protections, people may legitimately fear negative repercussions if they disclose LGBTQ+ identities in professional contexts. Existing research suggests the outcomes of queer identity disclosures are ambiguous, potentially increasing sense of belonging, job satisfaction and social support, while reducing stress and anxiety, yet simultaneously exposing the individual to increased heterosexism, discrimination, hostility and violence [10–15]. As a result, many LGBTQ+ workers engage in selective disclosure [16], making complex decisions about concealment/disclosure grounded in risk-benefit assessments that consider individual, interpersonal, and institutional factors [12, 15–18]. Based on an extensive literature review, Follmer and colleagues suggest “employees begin from a position of nondisclosure and then weigh the costs and benefits of disclosure before doing so” [3, p.173].

Introducing a journal special issue on identity concealment, Diane Quinn noted that “relatively little empirical research has explored the myriad factors that lead people to decide to conceal or reveal their identities” [8, p.231]. In the ensuing years, evidence has begun to amass regarding those influencing factors for LGBTQ+ disclosures in work contexts. At the individual level, identity centrality, internal identity positivity, identity perceptibility, impression management strategies, and anticipated outcomes of disclosure appear relevant [3, 12, 16]. Interpersonally, the presence of supportive colleagues and supervisors directly affects disclosure/concealment decisions [3, 12, 17]. Workers may selectively disclose to some coworkers and conceal with others, depending on their assessment of probable support; disclosure may be affected more by the presence of even one highly supportive colleague than by overall co-worker support [18]. In their review of disclosure literature, Follmer and colleagues note that having the support of colleagues can tip the scale in favour of disclosure even when institutions are themselves unsupportive [3]. At the institutional level, LGBTQ+ disclosure appears to be affected by organizational policies and rights protections, but even more so by professional culture and/or workplace microclimate [3, 12, 17]. Queer workers appear to be proficient in reading whether professed institutional protections will in fact prevent harm.

Other workplace factors that may affect identity disclosure/concealment decisions are just beginning to emerge in the literature. Employment type and status may influence decisions, such as permanent *versus* precarious employment [3], or working with children or groups vulnerable to harm [17]. Lloren and Parini found higher position in a workplace hierarchy improved mental health outcomes, though did not affect disclosures or experiences of heterosexism [13]. Disclosure may also be more likely after a professional reputation is well-established [3, 19]. Rengers et al. found disclosures were affected by how salient queer identity was in the workplace, how closely people engaged with co-workers and for how long, and whether interactions with clients/customers/patients were brief or extended [16]. There are hints that even in queer-friendly workplaces, disclosures to clients/customers/patients/students may be seen as ‘unprofessional’ and ‘inappropriate’ [19].

Identity perceptibility (the extent to which one is ‘read’ by others as queer) may be emerging both as influencing factor and as process. While the extent to which one is able to conceal queer identity affects disclosures, it also appears that it may be easier to disclose queer identity when in a relationship [16]; referring to a same-gender partner is simpler than making an identity announcement. At the same time, acceptance of LGBTQ+ workers may hinge on ‘homonormativity’–the extent to which gender expression and performance aligns with heterosexual gender roles and expectations, allowing assimilation [19, 20]. In some research, being queer was acceptable at work as long as people presented in accordance with heteronormative binary gender expectations: “In these workplaces, homonormativity meant being in a monogamous relationship (possibly with children), avoiding overt sexual talk or behavior, and dressing and acting normatively for their assigned gender” [19, p.1080]. Assimilation requires invisibility, not presenting as ‘too queer.’ On the flip side, visibly LGBTQ+ workers may be expected to embody heteronormative (binary) assumptions of queerness (the flamboyant gay man or the butch lesbian), with “certain kinds of performances required of them at work” [20, p.31] such that queer “visibility is often achieved through conformity to stereotypes” [20, p. 41]. In one study, workers avoided disclosing lest they be relegated to narrow perceptions of queerness [16].

Lastly, there are increasing admonitions that LGBTQ+ disclosure/concealment decisions take into account intersecting social identities. For example, Follmer and colleagues argue, “the study of disclosure decisions has largely ignored the ways in which multiple stigmatized identities intersect, thereby shaping disclosure processes” [3, p.178]. They urge researchers to consider, “how the intersection of identities influence not only individuals’ decisions to disclose but the outcomes of these decisions as well” [3, p. 178]. Pasek and colleagues also urge researchers to attend to how intersecting social identities affect disclosures, noting that there is some evidence to suggest LGBTQ+ disclosures may prove more beneficial for those from higher socioeconomic classes, and dominant racial/ethnic groups [15, p. 402]. Rengers and colleagues argue that at the individual level, disclosure decisions entail assessment of anticipated acceptance [16]–which surely would be complicated by the embodiment of multiple marginalized statuses.

### Keeping power at the center

Attention to intersectionality inherently means attention to social relations of power [21]. Intersectionality theory insists that each person lives at the nexus of multiple axes of power and oppression, such that some social identities grant privilege while others result in marginalization [22]. The experiences of (and risks faced by) a cisgender gay man will differ if he is white, able-bodied and higher socioeconomic class compared to if he is lower class, racialized and/or disabled. Disclosure decisions will inevitably be affected by multiple axes of potential marginality. Queer people who also face racism, ableism, classism, cissexism and/or ethnocentrism–even within queer communities and spaces–may not stand to gain the valuable social support from ‘similar others’ said to accrue from workplace identity disclosures [11].

Intersectional analysis, along with other critical approaches, insists on attending to “inequitable social systems and structures” [9, p.1321]. When attention to power relations is made central, an understanding of heterosexism emphasizes that microaggressions and other hostilities may be experienced differently depending on who the perpetrator is, the relationship between perpetrator and target, and the relative power relationships between the two [9]. For example, a heterosexist joke may be experienced very differently from a boss *versus* a co-worker. The relative power relationships include intersecting social identities, complicating vulnerability to heterosexism–and disclosure decision-making–through the embodiment of other forms of social privilege or oppression. While the environmental context, including workplace microclimate, supportive managers and co-workers, policies and protections can alter the experience of heterosexism and decisions about disclosures, that environment is complicated by power built into institutional hierarchies as well as embodied in social identities [9].

### Heteronormativity in the health professions

Finally, the degree of heteronormativity pervasive in a workplace and within a field of employment is an important factor in workplace LGBTQ+ identity concealment/disclosure. Heteronormativity (the belief that everyone is and should be heterosexual, the belief in heterosexuality and binary gender as normal and morally superior) remains persistent [23]. This ideology is at least as prevalent in the health professions as in any other social context. The professions are highly conservative environments, with considerable pressure toward conformity, and long histories of elitism [2, 24–26]. LGBTQ+ students and clinicians may face isolation, alongside erasure, invisibility and stereotyping in formal and informal curricula [2, 27–32]. Professional education and practice contexts are grounded in power hierarchies, making disclosure of a potentially stigmatizing identity a high stakes decision. In turn, when LGBTQ+ health professionals routinely conceal their identities to avoid reprisal, new entrants to the professions learn quickly that identity disclosure is unwise [1, 2, 27, 28, 30, 33–35]. This perpetuates the silencing effect of heteronormativity.

In this paper we examine the experiences of 13 LGBTQ+ health professionals (occupational therapists, nurses, physicians) from across Canada, asking how they engaged with identity concealment/disclosure and what factors influenced their strategic decision-making.

## Methods

This critical interpretive qualitative study explores experiences of 13 health professionals self-identifying as LBGTQ+. This included 6 occupational therapists, 5 physicians, and 2 nurses. The study received research ethics approval (Dalhousie University Health Sciences Research Ethics Board [REB] 2019–4733), after which participants were recruited using snowball sampling through research posters and social media from across Canada. To be eligible, participants required at least 5 years of practice experience in Canada in their professional field. Healthcare providers who responded had their eligibility confirmed and were provided with consent forms and study details.

Informed consent documents were sent to potential participants by email, then discussed at the beginning of each interview. Those who were interviewed in person signed a consent document, those who were interviewed by telephone provided verbal consent which was audio-recorded. Both strategies were approved by the REB. Once consent had been discussed, one of the three interviewers conducted a semi-structured interview with the participant by phone or in person. Each interview took between 60 and 90 minutes and explored experiences of marginality and belonging in professional contexts, including practice and training. Interviews were audio-recorded and transcribed verbatim. They were then checked for accuracy and any identifying information was removed. After multiple readings, transcripts were entered into ATLAS.ti, a qualitative data analysis software used to facilitate team coding and analysis. The codes used drew on theory and literature, with some also emerging from the data. Analysis moved between coded data and full transcripts and between data and theory, using an iterative approach. Once quotations had been organized, they were ‘cleaned’ by removing false starts and filler words such as ‘um’ and ‘ah’, to facilitate readability.

Team meetings, held weekly over many months, focused on interpretating the data using questions such as “What do you think this participant means when they say…?” We collectively pondered how we each were thinking about codes, whether they may need to be altered for greater nuance or accuracy, or whether new codes were needed [36]. These enabled us to use a form of ‘transpersonal reflexivity’ [37], during which perceptions, experiences, and beliefs become sources of insight through thinking collectively. Our weekly discussion gradually engaged more with data interpretation and theory. The specific framework of identity concealment/disclosure gradually emerged through team analysis and discussion, building on our existing understanding of heteronormativity and heterosexism. Together, these provide the overarching thematic structure for this paper.

Lived experience informed all aspects of this research, allowing us to mobilize our ‘biases’ and perspectives to enrich the analysis. Members of the research team included members identifying as LGBTQ+ and straight, who all have first-hand experience of some form of marginalization in their respective professions (occupational therapy, nursing, and medicine).

### Limitations

To minimize participant burden, only single interviews with participants were conducted. Additional interviews may have allowed participants to engage with deeper reflections, which is a limitation of this study. In addition, the inclusion of multiple professions enabled a focus on disclosure decision-making in heteronormative healthcare contexts broader than any one specific profession. However, this meant that there were smaller numbers of professionals from any given field, which may obscure profession-specific details. Thematic saturation is often linked to a notion of ‘information redundancy,’ which we certainly began to notice in later interviews. Yet we also agree with those who argue that saturation is a rather post-positivist stance, which inadequately captures the way interpretive analyses spiral ever deeper, uncovering layers of meaning and interpretation [36]. As we proceeded through analyses, we found the sample had sufficient conceptual density. Finally, while we examine the impact of intersecting social identities in this paper, it is limited by the fact that almost all of our participants were white.

## Results

To preserve confidentiality, we report only aggregated demographics (Table 1), and choose not to identify quotes by age, profession, or LGBTQ+ identity. We report elsewhere [38] on participants’ experiences of heterosexism and heteronormativity, and on their processes of calculating the risks and benefits of identity disclosures, which led most people to disclose selectively [4]. Here we sketch that context briefly, then detail the major themes for this analysis of the factors influencing concealment/disclosure: Degree of visibility; Practice context; and Power relations–including both professional power, and social power.

**Table 1:** Demographics.

Characteristic	Category	N = 13
**Age**	30s	7
	40s	4
	50s	2
**Years in practice**	5–9	6
	10–14	3
	15–19	2
	20+	2
**Location**	Rural/town	2
	Small/mid-sized city	6
	Large city	5
**Gender**	Men	4
	Women	7
	Fluid/non-binary	2
**Racialized**		1
**Disabled**		3
**Working class origins**		5

### Disclosing and concealing in heteronormative professional contexts

Participants described pervasive heteronormativity in these three health professions, often being the only openly LGBTQ+ person in their class or workplace, facing constant heteronormative questions and assumptions from colleagues and clients/patients in everyday conversations, and being warned by colleagues not to be ‘too visible’ in work contexts. The curricula in their educational programs ignored or stereotyped queer people, even as recently as 5 years prior to this study. Interpersonal heterosexist microaggressions were less common than the ubiquitous heteronormativity, though some people did face heterosexist comments and slurs, at least on occasion. It was particularly challenging when such hostility came from patients/clients, and addressing that directly was rarely supported by colleagues.

Not surprisingly, in that context most people concealed their queer identities, at least most of the time, and particularly with patients/clients. Most had gradually become more inclined to disclose as they proceeded through their careers, though two of the 13 never disclosed to anyone in professional contexts, and three others did so rarely, considering it inappropriate. With patients/clients, nine participants never or rarely disclosed, considering it a violation of professional boundaries; they typically only disclosed if they thought it was therapeutically beneficial. Even some who disclosed their LGBTQ+ identities to most if not all colleagues, continued to conceal with patients/clients, “at times hiding.” A few participants disclosed strategically, deciding when it was safe to challenge heteronormative assumptions. Only one person never concealed LGBTQ+ identity, displaying wedding and family photos to passively disclose to all colleagues and clients. But even that participant engaged in impression management, striving to look “less butch” at times, to better fit in professional contexts. Similarly, some men strove to appear “less gay.”

Assessments of disclosure risks and benefits were complex and ongoing. People concealed LGBTQ+ identities to avoid loss of professional status and credibility, therapeutic rapport with clients/patients, or respect from colleagues–or even the construction of a ‘tainted’ identity [39]. Some feared professional rejection and ostracism, while others sought to minimize potential harm: physical, emotional and professional. At the same time, people recognized that disclosing LGBTQ+ identity could foster therapeutic rapport, especially with some clients; they felt identity concealment left their engagements with patients overly superficial, to the point of raising ethical concerns. The benefit to queer clients/patients was particularly salient, and many participants found themselves much more able to advocate for others when they disclosed, no longer having to fear ‘stigma by association’ [40] if they spoke up about LGBTQ+ issues. Some thought identity disclosure was a kind of responsibility, acting as a role model for students, junior colleagues, and patients/clients.

Decisions to conceal or disclose queer identity as a health professional were shaped by risk-benefit calculations, which were influenced by the contextual factors we take up next: degree of visibility, practice context and power relations.

### Influencing factors: Degree of visibility

Participants had varying degrees of choice about whether or not to conceal their LGBTQ+ identities. Not everyone was able to pass as straight; those who were, faced more ongoing decision-making; those who were not, faced more ongoing risk of heterosexism. Most participants mentioned their self-perceived degree of queer visibility. One participant described herself as “an invisible lesbian,” saying, “Because I ‘look like a straight woman’… people just assume that you’re not a lesbian, when you look like me.” She disclosed selectively to colleagues and rarely to patients. Another participant described herself as “not a visible minority,” saying, “I just look like a possibly somewhat frumpy, 58-year-old white woman (laugh)… I don’t jump out. Like, I’m not particularly butch, I’m not particularly *not* butch, I just look like a regular gal.” She used invisibility to avoid disclosures she assessed as unsafe, and to virtually never disclose to clients. Another participant who never disclosed to patients and rarely to coworkers said she easily passes as cisgender and heterosexual because she appears stereotypically hetero-feminine: “It’s easy for me not to be out… I don’t fit that stereotype.”

Others were less able to conceal LGBTQ+ identities. One participant who was out everywhere felt conspicuous in professional contexts: “I look like a lesbian, I guess. (laugh) I feel like I do. I don’t have that same sort of look about me that a lot of other health professionals do, and I don’t think I look particularly feminine.” This was echoed by another participant who described herself as “somebody that could be easily identifiable as a lesbian”:

The stereotypes that gay and lesbians have, what they look like. Like, I’ve got a really short haircut. You know, my clothing, I guess, in some ways, could be seen as stereotypical lesbian, like the shoes I wear. (laugh) And just, maybe, some of my mannerisms and the way I talk.

She rarely disclosed at work, so had to deal with people mis-gendering her: “They have automatically assumed that I’m a guy… (laugh) so, that’s awkward.” She was always highly conscious of this when meeting new patients/clients. Another participant made some efforts with his appearance, yet still found clients read him as gay: “Sometimes when I talk with clients, I even noticed this today, a young guy I was working with actually, watching my hands move… I don’t think they’re used to hands moving.”

Degree of visibility was also affected by queer stereotypes, binary assumptions, and type of sexual identity. For example, one participant who was not easily able to conceal his identity, felt pressured by colleagues to present as the ‘right’ kind of gay, meeting stereotyped expectations.

With the girls at work, like, how do I explain? I don’t know. I’m definitely viewed as [Name] the Gay Guy. It’s not actually said, but I think it’s because I’m more flamboyant, you know? That’s expected of me. ‘Cause I fit the stereotypes in many ways… I find that’s just expected now, almost.

This obligatory identity display felt pressured and performative. Others found their sexual identities were unrecognized or poorly understood, making concealment a simpler choice. For example, a participant who identified as pansexual found when she had opposite-gender partners she was simply read as straight, erasing a pansexual identity that confounds simple binaries (straight or gay). Similarly, an asexual participant chose to simply never disclose in work contexts; the identity seemed too complicated to explain to colleagues, and without intimate partners disclosure was more difficult and felt unnecessary.

### Influencing factors: Practice context

Practice context affected identity concealment/disclosure in several ways. Participants suggested particular fields of practice facilitate or hinder disclosure: “Some disciplines are less accepting than others. So, if you want to do orthopaedics, you would never come out. If you want to do psychiatry, of course you would come out.” This was echoed by several others, with mental health practice seen as a relatively safe context to be out, along with academic settings, community settings and sexual health practice. One participant thought degree of risk connected with the degree of physical intimacy involved in practice:

Even now, depending on where you work, you want to be somewhat cautious about being fully out in your workplace. You know, patients may have issues. I would be concerned about allegations if you were doing, you know, intimate exams on people. Potentially, that would be problematic.

Relationships with colleagues also clearly mattered. One participant avoided disclosures with colleagues in a part-time job, where she never really got to know people. Others found disclosures far easier when surrounded by supportive colleagues, whether LGBTQ+ or not: “All of us who are diversity-focused.”

Patient/client population also affected identity concealment/disclosure. For example, working with vulnerable groups, such as in a corrections facility or with children, tended to hinder disclosure. Some concealed queer identities when working “with seniors and the elderly population sometimes, there’s certain stereotypes of them, that they have certain judgements.” Participants were also less likely to disclose when they only saw patients/clients for brief periods: “I don’t have to divulge much of my personal life, because I don’t have super close relationships with my clients.” When working with clients for only a few days rather than several weeks, “the investment or the consequence felt a little less.”

Finally, some participants saw identity disclosure as particularly challenging in small towns and rural areas, arguing that larger cities were relatively safer places to be out as professionals: “I might choose to work purposely in a setting where it’s a little bit more diverse… unfortunately I think it might be more of a metropolitan type of city.” As one participant said, “Being a more diverse city, there’s more acceptance for difference. So, I feel like I can be myself.” Some regions of Canada were also depicted as more conservative, carrying greater risk for disclosures. On the other hand, one participant thought it was even more important to be completely out in small towns, rural areas, and conservative regions, to give queer clients/patients someone to connect with.

### Influencing factors: Professional power relations

Decision-making about identity concealment/disclosure was clearly affected by positioning in hierarchies of professional power connected to career trajectory. Almost all participants spoke of becoming more likely to disclose queer identities as they progressed in their careers, gaining professional power. Students and medical residents were vulnerable to the judgements of numerous others with the power to influence their careers, significantly raising the stakes for identity disclosures. One participant, who was out throughout school and professional practice, nonetheless concealed queer identity during admissions, “because I was a little bit worried about that.” Another described identity concealment as a student “working with an older, white, religious male physician as my main preceptor” because “I didn’t feel like ostracizing myself.” Explaining selective identity disclosure, one participant noted, “When you’re not someone who has power over me I’m way more comfortable chatting about these things, *versus* letting someone know who I’m going to be asking for a future reference letter.”

Attaining licensure, job security and professional power granted some participants considerably more freedom:

When I started actually working in an attending role I was like ‘F*ck it, I’m just gonna be me’ and then, I’m at the point now that I don’t have that medical hierarchy crushing down on me. When you’re in the attending role, the puck stops with you, right? … I feel more comfortable to be myself, so then I started wearing a rainbow pen on my lanyard and I have pieces of rainbow tape wrapped around my stethoscope.

Nonetheless, this participant still thought earlier times of identity concealment were prudent: “I’d love to tell myself, ‘You know those times you hid or those times you weren’t honest, you could have been’… I’d love to tell myself that, but it was probably situationally appropriate.” Another participant passed as straight until well-established in career progression, fearing heterosexist repercussions. At the point of disclosure, “It was a non-issue. I had built up enough social equity within medicine, either because of my education work or my clinical work as well, that they didn’t see it as an issue.”

Interestingly, for some participants, *lack* of job security or career prospects also offered a certain freedom. One person had given up on potential promotions for various reasons, then found identity concealment far less important, because career risks were lessened. Similarly, a participant in a contract academic position described lower concealment stress: “‘Cause I’m contract academic staff, I’m not on a tenure track, I feel like I have more freedom.” In contrast, most of their colleagues concealed LGBTQ+ identities, saying, “‘Not until I get my tenure.”

### Influencing factors: Intersecting social power relations

Attaining professional power happens over time, so it is not always possible to tease apart the effects of career trajectory on concealment/disclosure *versus* the effects of aging and societal change over time. Age clearly had an influence, but the direction was inconsistent. For example, one participant who had attained considerable professional seniority before coming out in work contexts said, “I’m getting old enough that I figure, ‘What’s the worst thing that could happen?’ … [It’s made me] more courageous to be a more obvious advocate for LGBT issues.” Two participants who were older before they entered professional education said they were simply unwilling to conceal their identities, after being out for many years. They also noted that the heterosexism they encountered, particularly in school, might have been more damaging had they been younger:

It impacted me in that it p*ssed me off, but because I was a little bit older, I had been out for a really long time at that point and really confident in my identity, I don’t think it impacted in the way that it might have if I had been 20 or 21 and just kind of coming to my own identity.

In contrast though, one participant who was older, and completely out in all social spheres, chose to conceal queer identity upon entry to medicine, to avoid additional stigma: “I just didn’t need another mark against me. I already had enough. I was already quirky and older, and mouthy at times.”

This participant highlights how identity disclosure decisions take into account multiple intersecting social identities. Those that attach to social privilege may help mitigate the risks of disclosure, while identities that incur stigma and discrimination potentially amplify risk. The participants who concealed their queer identities in virtually all professional contexts, plus those who never disclosed to clients/patients and were highly selective in disclosing to colleagues all lived the intersection of multiple marginalized identities: they were women, from working class origins, experienced disability, non-binary gender identity, racialization and minority ethnicity. One described herself as among “the mavericks of medicine, just because otherwise, I don’t easily fit the kind of usual, which puts me at a lot of risk.” Another participant said about rarely disclosing, “Culturally as well, it’s like, ‘That’s none of your business!’”

If we placed participants on a continuum, from complete concealment to complete disclosure, and on a continuum from least to greatest social privilege, the ordering of the two would map onto each other closely, though imperfectly. Several people referred to themselves as privileged when discussing their ability to be out in professional contexts. Those who were quite consistently out tended to be white, able-body-minded, cisgender men and women from higher social class backgrounds. Two white, able-body-minded, cisgender men from working-class origins were also quite out in professional contexts, though rarely with clients/patients. Visibility of course complicated this, as not everyone was fully able to conceal queer identity.

Interestingly, while privilege attached to membership in socially dominant groups could ameliorate the risks of queer identity disclosures, making it relatively safer for participants with the most privilege to be out, there was one counter instance that suggests loss of privilege can pose its own form of risk. One participant was a white, able-body-minded man, from the higher-class background traditional in medicine, older, with considerable professional seniority. Yet he concealed his gay identity for decades, in all spheres of his life. Before coming out in professional contexts, he feared tremendous rejection, loss of position and status. While social power, privilege, may protect LGBTQ+ health professionals somewhat from the harms of disclosing a stigmatized identity, it also seems possible that those who experience privilege in every other aspect of their lives experience the potential loss of (or harm to) privileged social status through disclosure of a stigmatized identity as a much more substantial risk, never having lived with other forms of oppression.

## Discussion

Surrounded by pervasive heteronormativity in health professions educational and practice contexts, participants engaged in ongoing risk-benefit assessment to inform their decisions about LGBTQ+ identity concealment/disclosure. Decision-making was influenced by individual, interpersonal, environmental factors, as well as layers of power relations. The extent to which queer identity was visible (‘identity perceptibility’) pushed some people to navigate disclosure whether they wanted to or not–they were ‘read’ as queer. Their focus then became impression management, controlling their presentation of self to avoid being locked into unwanted stereotypes [3, 18, 19]. Notably, the participant who was arguably least able to pass as straight found himself caught in colleagues’ stereotypes of flamboyant gay men, constructing command performances of queerness [20].

Homonormativity (closeness to approximating hetero-expectations) seems to also influence individual visibility. Those who were least open in professional contexts were those who lived outside normative queer identities, as asexual, pansexual, gender fluid or gender non-binary. None of our participants reported having unconventional relationships (such as polyamory, or open relationships) which might have proven challenging to assimilate into professional heteronormativity. People deliberately referred to same-gender husbands and wives to “normalize” queer realities. Some studies of ‘gay-friendly’ workplaces suggest silence regarding sexual and gender non-conformity is a prerequisite for tolerance [20]. As Kelly and colleagues note, this privileges the “respectably queer,” leaving out “those who cannot, or choose not to, assimilate into mainstream straight culture” [19, p.1079]. They quote one of their study participants saying, “It is an act of courage to present myself the way that I desire without censoring myself” [19, p.1085].

Practice context affected disclosures through a mix of factors: professional microculture, workplace microclimate, interpersonal interactions with colleagues, and patient/client population. Some fields of practice were seen as more or less amenable to queer health professionals, depending in part on how ‘hands on’ the work is, and the degree of vulnerability among patients/clients [16, 17]. Clearly the presence of supportive colleagues matters [3, 12, 17, 18]–though they need not be LGBTQ+, but possibly just ‘diversity minded.’ The investment of emotion and energy into disclosures may also have differing ‘pay off’ depending on whether contact with co-workers and clients/patients is fleeting or ongoing. That consideration may not, however, predict the decision. Some people may not bother disclosing when they will only work with someone briefly, others may read the situation as low-risk, feeling freer to disclose if they will never see the person again [16].

The hints in recent literature about disclosure being influenced by employment type and status [3, 13] were borne out in our study, though again the decisional outcome is not pre-determined. As Follmer and colleagues suggest, “permanent *versus* contingent status in an organization might influence disclosure decisions… [with] greater likelihood of concealment when interacting with a person who maintained power or influence over them in the organization” [3, p.175]. Some of our participants were very clear that disclosure was made possible when they attained full professional licensure, status and authority, no longer at the mercy of higher-powered others. Attending physicians were among the most open about their LGBTQ+ identities, while the two least open were just finishing medical residencies. One person delayed any disclosures till well into their career, once professional respectability was well-established [19]. On the other hand, at least one person felt free to disclose because their employment was inherently precarious, with little at stake.

Finally, though the pattern was only suggestive, it appears that intersections with other privileged or oppressed social identities affect LGBTQ+ disclosures. Those most likely to disclose in most work contexts tended to be white cisgender men, plus one white cisgender woman who had been out for many years prior to professional education. The participants *least* likely to disclose at work lived with multiple marginalized identities due to mental health status, disability, age, gender, social class background, ethnicity, racialization and non-binary gender identity. In reviewing literature about identity concealment, Follmer and colleagues note, “researchers are encouraged to consider why maintaining multiple stigmatized identities is likely to influence disclosure decisions and to consider how specific identity characteristics might interact to influence the decision to disclose or not disclose” [3, p.178]. We cannot address that in detail from our data, though one participant spoke of already having too many marks against them to disclose queer identity. If “power plays a role in shaping the processes that contribute to different life experiences and outcomes for people at different intersections” [21, p.222], the risks to professional status and credibility will vary accordingly. There are hints in our data that this is a promising direction for further inquiry. For those experiencing multiple forms of oppression–classism, sexism, racism, ethnocentrism, ableism, sanism–disclosing a concealable identity that may bring hostility, trauma, and harm is a very different decision than it is for those who experience social privilege on multiple levels [9]. There is also something intriguing in our data about the way potential loss of privilege may hinder LGBTQ+ identity disclosure. This may connect with the way some fields of practice seemed least open to queer professionals; within each profession the less prestigious specialties appeared to be seen as most queer-friendly.

Taking all those factors together, what becomes very clear is the complexity of LGBTQ+ disclosure decision-making in the health professions. Some participants appeared to be heavily influenced by their degree of visibility/invisibility, others by job security and professional status, by the practice context and professional culture as well as type of client/patient contact, and/or by the potential loss of privilege or compounding of multiple axes of oppression. If the effect of disclosure is potentially risky, and the extent of risk is connected to the professional power relationships between people in the work context [9], the health professions’ being mired in power hierarchies amplifies the stakes for disclosures [1, 2, 24, 25, 27–35]. Non-disclosure or strategic selective disclosure seem to be employed by health professionals to reduce risk in the context of power hierarchies. As Williams and colleagues note regarding disclosure/concealment and homonormativity/non-conformity, “In the workplace, where livelihoods are at stake, it makes little sense to blame individuals for making one of these two equally fraught choices” [20, p.42]. The almost-obligatory disclosure rhetoric [e.g., 11, 14] may be short-sighted, failing to acknowledge the complexity of strategic decision-making ongoing for health professionals given the power hierarchies within which they work.

The almost automatic resistance to the idea of disclosures to patients/clients in the name of ‘professionalism’ suggests that concept needs interrogation. It appears to be grounded in heteronormativity and a perception of LGBTQ+ identities as problematic, too private (shameful?) to disclose with clients/patients. Meanwhile straight health professionals embody their sexual and gender identities often very visibly, with no concern that they are violating professional boundaries. Some participants were explicitly taught (and taught others) that sharing their queer identities with patients/clients is unprofessional, yet heterosexual professionals may freely chat about their families, weekend activities and so on. This is heteronormativity in action [10]. Drawing on Mizzi’s notion of “heteroprofessionalism,” [41] Davies and Neustifter suggest notions of ‘professionalism’ are leveraged through accreditation and discourses of standards, assessment, accountability and appropriateness to regulate and discipline queer professionals’ embodiments of sexual and gender identities [42]. Heteroprofessionalism in the health professions silences and erases diversity in the name of conformity.

### Implications

A small qualitative study makes no claims to generalizability, but our results support important directions for further research. Clearly identity concealment/disclosure at work is a complex matter, and our results suggest the importance of attending to social factors on multiple levels, holding them as at least as significant as individual attributes like ‘reaction sensitivity,’ self-esteem and personal positive regard [e.g., 43–45]. In particular, attention to type of employment, power hierarchies at work, and power relations resulting from intersecting social identities appear promising. Though research in these areas is nascent, there are also possible practice implications for those working to support LGBTQ+ people struggling with identity concealment/disclosure. Recognizing the skill involved in risk-benefit calculations, and helping people tease out the multiple levels of influencing factors may help clarify decision-making.

Within the health professions and specific workplaces, it is clear that non-discrimination policies, which are visible and enforced, can help create a safer culture [13, 46]. Given the power hierarchies institutionalized in the health professions, however, it is important to remember that people will be unlikely to utilize anti-discrimination policies without ongoing encouragement and protections in place [17]. Workplace/professional culture is also improved by the visibility of supportive colleagues and senior leaders, as well as ongoing education regarding inclusion [18, 46]. People can learn to challenge heterosexism. Educators in the health professions can model strategic approaches to inviting disclosures from trainees, clearly signalling safety, and can shoulder responsibility for intervening in heterosexist interactions, working with trainees to find preferred approaches to intervention [47]. Mentoring programs and peer support groups can also help establish a professional culture wherein heterosexism and heteronormativity are disrupted [46, 48]. In part this provides immediate support and guidance, but it can also begin to shift cultural norms and expectations [49, 50]. It is clearly important that intersecting identities of privilege and oppression be taken into account in setting up mentoring and peer support. Finally, it is key that full belonging within the health professions not be predicated on assimilation, a homonormative approximation of heteronormative expectations that renders queer professionals effectively invisible [19, 20]. Rather, the heteronormative cultures of the health professions need queering.

## Conclusions

The literature suggests that a myriad of factors affect LGBTQ+ identity disclosure, which was evident among our participants. In a work context steeped in historical and current hierarchies of power, the queer health professionals in this study suggest deciding whether or not to disclose is affected by how different types of professional hierarchies–interpersonal, intraprofessional, and institutional–intersect with their multiple social identities. What may be appraised as a safe or strategic disclosure to some may pose great risk to others when considering factors like professional experience, job security, and reliance on others for career advancement. Workplace contextual factors including patient/client population and practice area also influence perceived and real risks of disclosing. Heteronormativity is enforced in the health professions by mobilizing normative power, regulating the behaviour of queer members. This research highlights the need to consider power from an intersectional perspective to understand the individual and situational factors considered when deciding whether or not to disclose LGBTQ+ identity, as well as the need to disrupt systemic heteronormativity.
